# LINC00963 Promotes Cancer Stemness, Metastasis, and Drug Resistance in Head and Neck Carcinomas via ABCB5 Regulation

**DOI:** 10.3390/cancers12051073

**Published:** 2020-04-26

**Authors:** Shiao-Pieng Lee, Pei-Ling Hsieh, Chih-Yuan Fang, Pei-Ming Chu, Yi-Wen Liao, Chuan-Hang Yu, Cheng-Chia Yu, Lo-Lin Tsai

**Affiliations:** 1 School of Dentistry, National Defense Medical Center, Taipei 11490, Taiwan; shiao-pieng@yahoo.com.tw; 2 Department of Dentistry, Tri-Service General Hospital, Taipei 11490, Taiwan; 3 Department of Anatomy, School of Medicine, China Medical University, Taichung 404, Taiwan; plhsieh@mail.cmu.edu.tw (P.-L.H.); pmchu@mail.cmu.edu.tw (P.-M.C.); 4 School of Dentistry, College of Oral Medicine, Taipei Medical University, Taipei 110, Taiwan; 100044@w.tmu.edu.tw; 5 Division of Oral and Maxillofacial Surgery, Department of Dentistry, Wan Fang Hospital, Taipei Medical University, Taipei 116, Taiwan; 6 School of Dentistry, Chung Shan Medical University, Taichung 40201, Taiwan; rabbity0225@gmail.com (Y.-W.L.); tao2008@csmu.edu.tw (C.-H.Y.); 7 Department of Dentistry, Chung Shan Medical University Hospital, Taichung 40201, Taiwan; 8 Institute of Oral Sciences, Chung Shan Medical University, Taichung 40201, Taiwan

**Keywords:** head and neck, lncRNA, LINC00963, cancer stemness, chemoresistance, ABCB5

## Abstract

Accumulating studies have indicated that long non-coding RNAs (lncRNAs) participate in the regulation of cancer stem cells (CSCs), which are crucial in tumor initiation, metastasis, relapse, and therapy resistance. In the current study, RT-PCR analysis was employed to evaluate the expression of LINC00963 in tumor tissues and oral CSCs. Stemness phenotypes and the expression of CSCs markers in oral cancer cells transfected with sh-LINC00963 were examined. Our results showed that the expression of the lncRNA LINC00963 was up-regulated in oral cancer tissues and CSCs. We found that the downregulation of LINC00963 inhibited CSC hallmarks, such as migration, invasion and colony formation capacity. Moreover, suppression of LINC00963 reduced the activity of stemness marker ALDH1, the percentage of self-renewal, chemoresistance and the expression of multidrug-resistance transporter ABCB5. Most importantly, we demonstrated that knockdown of LINC00963 decreased self-renewal, invasion and colony formation ability via ABCB5. Analysis of TCGA (the Cancer Genome Atlas) datasets suggested that the level of LINC00963 was positively correlated with the expression of the cancer stemness markers (Sox2 and CD44) and drug resistance markers (ABCG2 and ABCB5). Altogether, our results showed that suppression of LINC00963 may be beneficial to inhibit chemoresistance and cancer relapse in oral cancer patients.

## 1. Introduction

Head and neck cancers (HNC) represent one of the common neoplasms worldwide, and the majority of HNC are squamous cell tumors found in the oral cavity and larynx. Among patients with HNC, men accounted for two-thirds of these cases [1] and their prognosis is often poor as regional recurrence and distant metastasis after treatment remain prevalent [2,3]. To overcome this obstacle, several attempts have been made by researchers to target cancer stem cells (CSCs), which possess both self-renewal and differentiation capabilities [4]. CSCs have been considered to be responsible for the cancer cells evading or coping with the presence of therapeutics [4]. In oral cancer, several approaches have been employed to enrich and isolate CSCs, such as sphere formation [5], cell surface expression of specific stem cell markers (e.g., CD44) [6], increased enzymatic activity (e.g., aldehyde dehydrogenase, ALDH1) [7] or efflux of vital dyes by multi-drug transporters (e.g., ABC transporters) [8]. In order to eliminate CSCs, further studies are necessary to identify the oncogenic factors that drive cancer stemness. 

Accumulating evidence has indicated that non-coding RNAs, such as microRNAs (miRs; around 21–24 nucleotides) or long non-coding RNAs (lncRNAs; longer than 200 nucleotides), can regulate malignant phenotypes and CSCs features [9]. Unlike microRNAs that bind to the 3′ untranslated region (UTR) of target mRNAs post-transcriptionally, lncRNAs modulate CSC properties through various molecular levels, such as via epigenetic, transcriptional and post-transcriptional regulation [10]. Owing to the potential to modulate tumor-associated characteristics, non-coding RNA-related therapeutics that are specifically aimed at CSCs have been assessed in a variety of studies [11,12,13]. A better understanding of the roles of lncRNAs in oral CSCs may contribute to the development of a therapy that abrogates tumor growth or sensitizes chemotherapeutic agents.

Long intergenic non-coding RNA 963 (LINC00963;MetaLnc9) has been identified as the lncRNA that was involved in the progression from androgen-dependent to androgen-independent prostate cancer through physical association with an epidermal growth factor receptor [14]. LINC00963 enhanced the migration and invasion of lung cancer cells in vitro and metastasis in vivo, and has been found to interact with phosphoglycerate kinase 1, leading to activation of the oncogenic AKT/mTOR signaling pathway [15]. It has been shown that LINC00963 promoted carcinogenesis and radioresistance in breast cancer as LINC00963 was able to antagonize the repressive activity of miR-324-3p on activated CDC42 kinase 1 expression [16]. Another study showed that LINC00963 served as a sponge for miR-214-5p to contribute to the progression of esophageal squamous cell carcinoma [17]. Since LINC00963 participated in the aggressiveness in various cancers, it is of great importance to elucidate whether LINC00963 possess the ability to modulate cancer stemness, especially in oral cancer.

In the present study, we assessed the expression of LINC00963 in oral squamous cell carcinoma (OSCC) and examined the contribution of LINC00963 in cancer stemness. We also evaluated the significance of LINC00963 in chemosensitivity and its downstream factor. These results provided insight into the oncogenic role of LINC00963 in OSCC.

## 2. Results

### 2.1. LINC00963 Is Upregulated in the Specimens of Oral Cancer and Oral CSC

**Figure 1:**
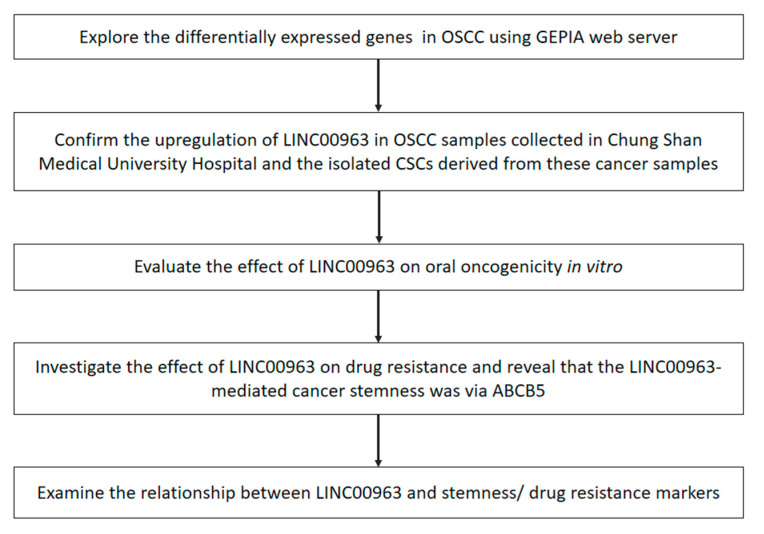
The flowchart of the experimental design.

**Figure 2:**
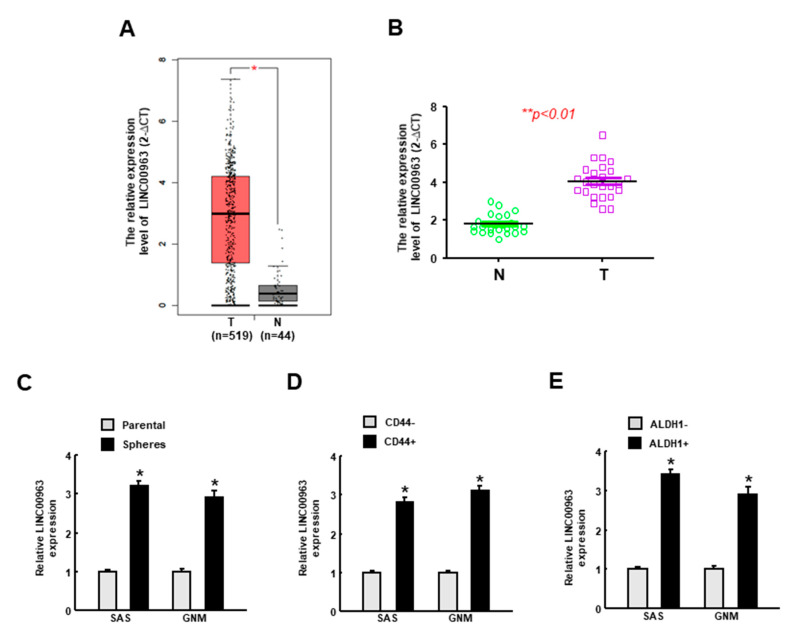
The expression of LINC00963 is upregulated in the oral cancer tissues and cancer stem cells. (**A**) Analysis of the expression level of LINC00963 in normal and oral squamous cell carcinoma (OSCC) tissues using TCGA dataset; (**B**) verification of the upregulated LINC00963 in OSCC tissues compared to normal counterparts (*n* = 25 for each group) using RT-PCR analysis; expression of LINC00963 in spheroid (**C**), CD44^+^ (**D**), and ALDH1^+^ (**E**) cells were evaluated by RT-PCR. Experiments were repeated three times and representative results were shown. Results are means ± SD of triplicate samples from three experiments. * *p* < 0.05.

To identify crucial lncRNAs in OSCC tumorigenesis, we generated this flowchart to elaborate the experimental design (Figure 1). Intially, to explore the differentially expressed genes in oral carcinomas, we used the Gene Expression Profiling Interactive Analysis (GEPIA) online database (http://gepia.cancer-pku.cn) and identified higher expression of LINC00963 in tumor tissues (Figure 2A). To verify that LINC00963 was upregulated in clinical samples, we used real-time qRT-PCR to examine the OSCC specimens that were collected in our hospital and confirmed that the expression of LINC00963 in OSCC tissues was indeed elevated (Figure 2B). Moreover, the relative expression of LINC00963 was consistently increased in sphere cells (Figure 2C), CD44+ (Figure 2D), and ALDH1+ (Figure 2E) cells derived from two types of OSCC cell lines (SAS and GNM), suggesting that LINC00963 was also elevated in CSCs and may be associated with the cancer aggressiveness.

### 2.2. Silencing LINC00963 Inhibits the Tumorigenicity of Oral Cancer Cells

**Figure 3:**
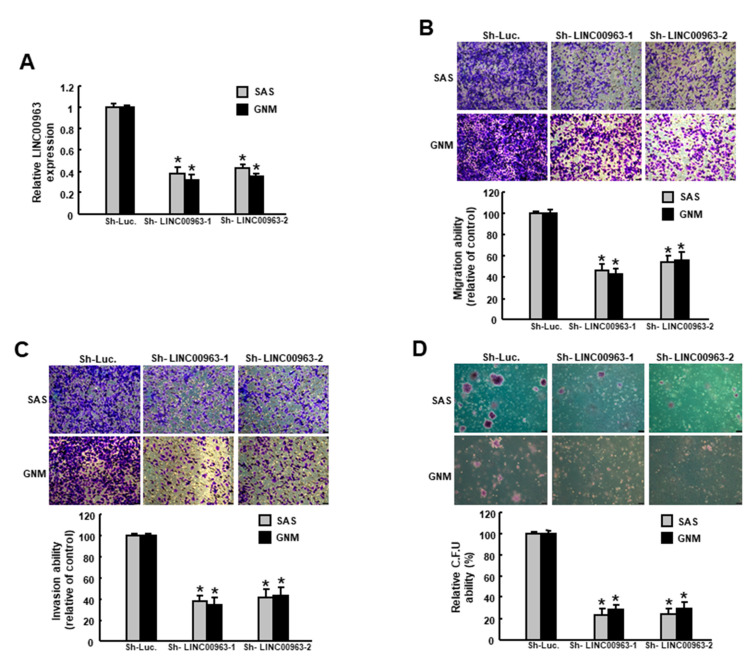
Inhibition of LINC00963 suppresses the stemness phenotypes in OSCC. (**A**) Cells were transfected with lentiviral vectors to inhibit LINC00963; (**B**) migration, (**C**) invasion, and (**D**) colony formation capacities were evaluated. Experiments were repeated three times and representative results were shown. Results are means ± SD of triplicate samples from three experiments. * *p* < 0.05.

**Figure 4:**
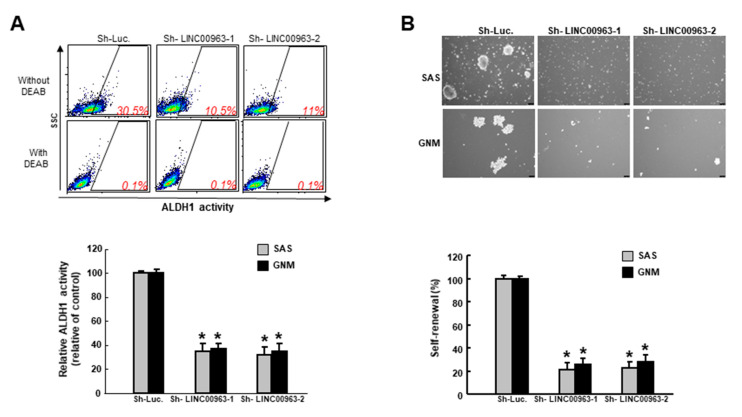
Downregulation of LINC00963 reduces the cancer stem cells (CSC) marker and self-renewal ability in OSCC cells. (**A**) The ALDH1 expression was analyzed by flow cytometry and (**B**) self-renewal capacity was assessed by serial sphere formation assay. * *p* < 0.05.

To evaluate the effect of LINC00963 on the features of cancer stemness, we utilized the lentiviral-mediated approach to downregulate the expression of LINC00963 in two types of oral cancer cells (Figure 3A). As expected, the ability of migration (Figure 3B), invasion (Figure 3C) and colony formation (Figure 3D) were all decreased with LINC00963 knockdown. In addition, the proportion of ALDH1-expressing cells was reduced following the transfection of LINC00963 lentivirus (Figure 4A), indicating that suppression of LINC00963 may downregulate the existence of CSCs. In associated with this observation, we showed that knockdown of LINC00963 inhibited the self-renewal capacity of these two OSCC cell lines (Figure 4B). Taken together, these findings confirmed that the modulation of LINC00963 could repress the oral oncogenicity in vitro.

### 2.3. LINC00963 Modulates Chemosensitivity of Oral Cancer through ABCB5

**Figure 5:**
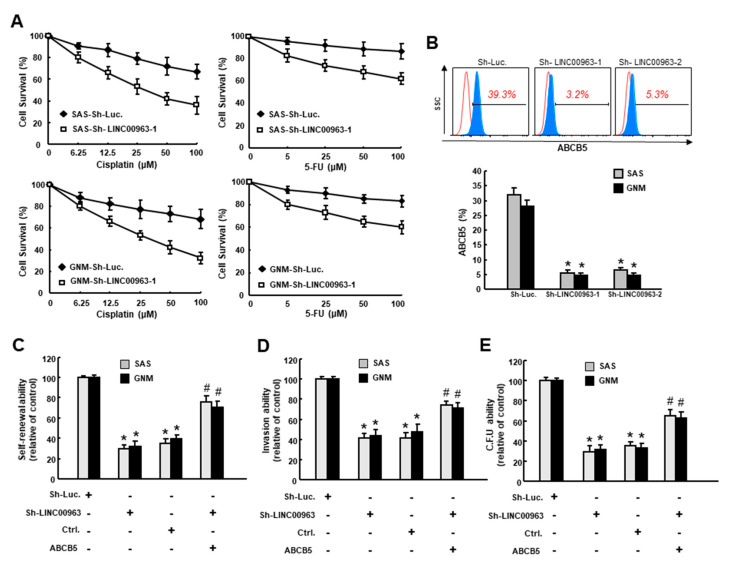
The silence of LINC00963 sensitizes chemotherapy and downregulates CSC features through suppression of ABCB5. (**A**) Cell survival of OSCC cells transfected with sh-Luc or sh-LINC00963 in response to Cisplatin (left) and 5-FU (right) treatment; (**B**) proportion of ABCB5-expressing OSCC cells transfected with sh-Luc or sh-LINC00963 using flow cytometry; overexpression of ABCB5 rescued the inhibited (**C**) self-renewal, (**D**) invasion, and (**E**) colony-forming abilities in cells transfected with sh-LINC00963. Experiments were repeated three times and representative results were shown. Results are means ± SD of triplicate samples from three experiments. * *p* < 0.05 sh-LINC00963 group compared to the sh-Luc group. # *p* < 0.05 ABCB5 overexpression+sh-LINC00963 group compared to sh-LINC00963 group.

Given that chemoresistance has been attributed to cancer stemness, we sought to investigate whether inhibition of LINC00963 could enhance the chemosensitivity of oral cancer. We chose the commonly used chemotherapy drug cisplatin (CDDP) and 5-Fluorouracil (5-FU) and treated SAS and GNM cells with various concentrations of cisplatin (0–100 μM) followed by an examination of MTT assay. As shown in Figure 5A, oral cancer cells with sh-LINC00963 exhibited lower resistance to cisplatin or 5-FU compared to sh-Luc control. Moreover, the percentage (Figure 5B) and protein expression level (Supplementary Figure S1) of ABCB5 (ATP-binding cassette, subfamily B (MDR/TAP), member 5) was significantly reduced in both SAS and GNM cells with sh-LINC00963 knockdown. As an ATP-binding cassette transporter, ABCB5 has been known to act as a drug efflux transporter and confer multidrug resistance in diverse malignancies [18,19]. In OSCC, ABCB5 also has been considered as a putative CSC compartment and was associated with tumor progression [8]. In line with these findings, our results demonstrated that inhibition of LINC00963 downregulated the chemoresistance and the expression of ABCB5. Most importantly, we showed that the repressed self-renewal (Figure 5C), invasion (Figure 5D), and colony-forming (Figure 5E) capacities after a silence of LINC00963 were all reversed by overexpression of ABCB5 (Figure 5C–E), suggesting that LINC00963-mediated cancer stemness was through regulation of ABCB5.

**Figure 6:**
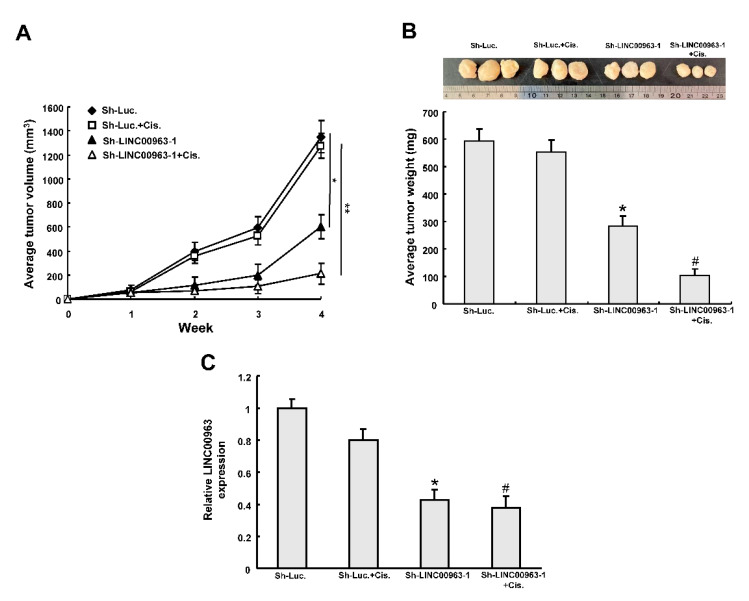
LINC00963 knockdown enhances chemosensitivity in mice. Tumor volume (**A**) and the tumor weight (**B**) was measured after injection of SAS-CSC treated with indicted treatment. (**C**) The relative expression of LINC00963 expression of in the excised tumors. * *p* < 0.05 sh-LINC00963 group compared to the sh-Luc group. # *p* < 0.05 sh-LINC00963+Cis. group compared to sh-Luc.+Cis. group. Define.

To further investigate whether the LINC00963 cisplatin sensitivity of OSCC cells in vivo, we subcutaneously injected four groups of stably transfected cells into xenografts. As shown in Figure 6A,B, LINC00963 knockdown attenuated xenograft growth and weight and this effect was enhanced by cisplatin treatment. The expression of LINC00963 was decreased following LINC00963 knockdown in the excised tumor tissues (Figure 6C).

**Figure 7:**
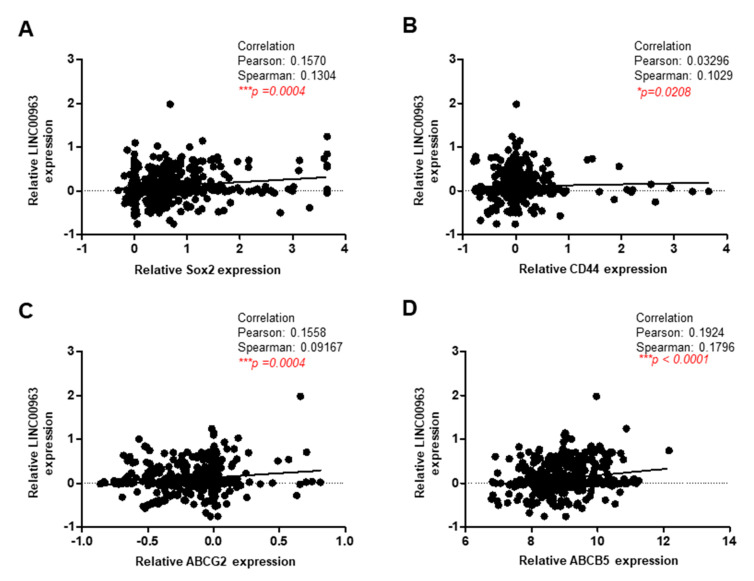
LINC00963 is positively correlated with various stemness and drug resistance markers. The relationship between LINC00963 and several CSC-associated molecules, including stemness markers (Sox2 and CD44) and multidrug resistance markers (ABCG2 and ABCB5), were evaluated through a TCGA dataset using Pearson’s correlation.

**Figure 8:**
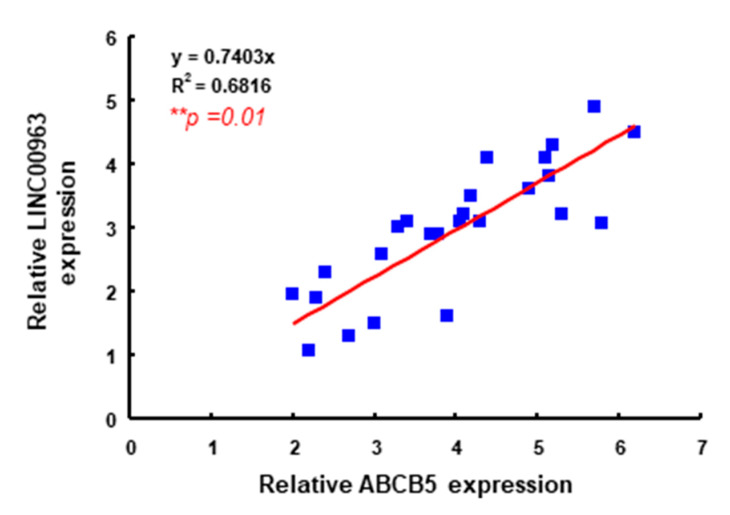
A positive correlation between LINC00963 expression and ABCB5 in Taiwanese OSCC specimens by qRT-PCR and linear regression analysis. ** p < 0.01.

Furthermore, we analyzed the OSCC data from the Cancer Genome Atlas (TCGA) and found that the expression of LINC00963 was positively associated with cancer stemness (Sex determining region Y-box 2; Sox2 and CD44) and drug resistance (ABCG2 and ABCB5) molecules (Figure 7), which was consistent with the aforementioned findings. Here, we further validated the results by analyzing ABCB5 and LINC00963 expression in Taiwanese OSCC specimens. As shown in Figure 8, LINC00963 is positively correlated with ABCB5 expression in Taiwanese OSCC specimens. Collectively, we demonstrated that LINC00963 was related to several CSC-associated factors and exerted its oncogenic capacity via ABCB5.

## 3. Discussion

Over the past few decades, the greatest barrier to effective cancer treatment has been cancer stemness. It is becoming increasingly clear that lncRNAs play central roles in the regulation of CSCs, which have been known as the key to cancer relapse and drug resistance. Nevertheless, current knowledge on the significance of LINC00963 in oral CSCs is still limited. In the current study, we found that the expression of LINC00963 was overexpressed in OSCC tissues. Particularly, the relative expression of LINC00963 was elevated in oral CSCs. Numerous studies have revealed that higher expression of LINC00963 was correlated with lymph node metastasis, TNM stage and shorter overall survival in several cancers [17,20], suggesting that LINC00963 may possess oncogenic activities and contribute to tumor progression. Indeed, it has been revealed that increased LINC00963 promoted tumorigenesis and facilitated metastasis in a variety of cancers [16,17] and suppression of LINC00963 downregulated proliferation, propagating capacities and induced apoptosis of cancer cells [17,20]. In agreement with these findings, we showed that inhibition of LINC00963 repressed several stemness parameters, including aggressive features and self-renewal capacity. Our findings indicated that LINC00963 may be a potential treatment target to diminish metastasis and cancer recurrence. These effects may be due to the impact of LINC00963 on oral CSCs, which was supported by our results showing downregulation of LINC00963 suppressed the proportion of ALDH1-expressing oral cancer cells. 

It has been known that the effectiveness of chemotherapy for OSCC is frequently restricted due to multidrug resistance. In the recent study, we demonstrated that the silence of LINC00963 enhanced the chemosensitivity and reduced the cell survival of oral cancer cells. To date, studies regarding the effect of LINC00963 on drug resistance are still lacking. One of the previous studies has shown that knockdown of LINC00963 sensitized breast cancer cells to radiation via the induction of DNA damage and oxidative stress [16]. Our results proved that approaches to downregulate LINC00963 could serve as adjuvant agents to improve treatment effects as the cancer cell survival was dramatically reduced. We demonstrated that the expression of ABCB5 was reduced in oral cells transfected with sh-LINC00963. ABCB5 is a member of the human P-glycoprotein family and has been revealed to regulate the fusion, growth, and differentiation of progenitor cells [21]. Besides, ABCB5 was critical to tumor vascular invasion and the ABCB5-dependent metastatic propensity was found to be dependent on IL-8/AXL signaling [18]. As a drug efflux transporter, it has been shown that ABCB5 mediated anticancer drug resistance [19,22,23] and conferred clinically relevant chemoresistance to CSCs in human colorectal cancer [18,24]. In OSCC, we showed that the enhanced chemosensitivity after a silence of LINC00963 was mediated by a downregulation of ABCB5. These results also coincided with a positive correlation between LINC00963 and another drug resistance marker, ABCG2, in OSCC data from TCGA. 

According to our findings, ABCB5 may not just participate in the drug resistance of OSCC, it is also involved in the LINC00963-mediated CSCs regulation as overexpression of ABCB5 preserved the inhibited self-renewal, invasive ability and colony-forming capacity caused by knockdown of LINC00963. Several studies have indicated that ABCB5 was able to identify CSC in diverse human malignancies [22,23], and confer CSC maintenance through an IL-1β/IL-8/CXCR1 cytokine signaling circuit to keep slow-cycling [25]. It has been shown that high ABCB5 expression was significantly related to tumor formation, metastasis and recurrence in OSCC and ABCB5 was co-labeled with CD44 (oral CSC marker) [8], suggesting ABCB5 was associated with oral CSCs and multistep carcinogenesis of OSCC [8,26]. It has been demonstrated that ABCB5 can function as an independent prognostic factor of OSCC [8]. A recent review article has included ABCB5 as one of the CSCs biomarkers in HNC [27]. Other commonly used biomarkers, such as Sox2, CD44, were also found to be correlated with clinical characteristics of HNC, such as staging, tumor size and lymph node metastasis [27]. Our analysis of the TCGA dataset revealed that LINC00963 was positively related to Sox2 and CD44, which was consistent with the finding that LINC00963 could regulate CSCs. 

One of the mechanisms underlying the cancer regulation of lncRNAs was that lncRNAs could act as molecular decoys to sequester miRNAs and downregulate the suppressive effect of their target genes [28]. It has been shown that LINC00963 functioned as a competitive endogenous RNA for miR-214-5p/RAB14 [17] in esophageal cancer, miR-625/ HMGA1 [20] or miR-324-3p/ ACK1 [16] axises in breast cancer. LINC00963 also has been reported to exert its oncogenic potential through the Akt pathway [14,15]. Previously, it has been shown that inhibition of the Akt pathway increased the pool of melanoma stem cells through enhancing ABCB5 functionality [29]. Since our data suggested that LINC00963 contributed to the aggressiveness and chemoresistance of oral cancer via the downregulation of ABCB5, it is plausible to speculate that Akt may play a role in the LINC00963-regulated ABCB5 expression. Further studies are needed to confirm this hypothesis.

In summary, we demonstrated that the aberrant upregulation of LINC00963 in OSCC contributed to the cancer stemness. Suppression of LINC00963 effectively reduced the cancer aggressiveness and sensitized chemotherapy through the downregulation of ABCB5. These data indicated that inhibition of LINC00963 may serve as a potential approach to eliminate CSCs and be useful for chemoprevention.

## 4. Materials and Methods

### 4.1. OSCC Tissues and Cell Culture

OSCC (T) and normal paired noncancerous tissues (N) were collected after obtaining written informed consent. All procedures were conducted in accordance with the Declaration of Helsinki, and approved by The Institutional Review Board in Chung Shan Medical University Hospital (approval code: CSMUH No:CS18043). The clinical characteristics of OSCC tissues in Supplementary Table S1. Tumor tissues were snap-frozen in liquid nitrogen and stored at −80 °C for quantitative real-time reverse transcription–PCR (qRT-PCR). The OSCC cell lines SAS (tongue squamous cell carcinoma) and GNM (gingival carcinoma neck metastasis) cells were cultivated as previously described [30]. Isolation of ALDH1^+^ or CD44^+^ oral CSCs were conducted by staining cells with ALDEFLUOR assay kit (StemCell Technologies, Inc., Vancouver, BC, Canada) or CD44 antibody followed by sorting with FACSAria II cell sorter (BD Biosciences, San Jose, CA, USA).

### 4.2. qRT-PCR Analysis

qRT-PCR was performed using TaqMan assay with a specific primer (Applied Biosystems, Carlsbad, CA, USA) and detection was conducted using a StepOne Plus real-time PCR system [31]. Total RNA was prepared from cells using Trizol reagent according to the manufacturer’s protocol (Invitrogen, Carlsbad, CA, USA). qRT–PCRs of mRNAs were reverse-transcribed by the Superscript III first-strand synthesis system (Invitrogen) and reactions on the resulting cDNAs were carried out on an ABI StepOne™ Real-Time PCR Systems (Applied Biosystems). The primer sequences used in this study were listed as follows: LINC00963: 5′-ATAAGCAAAGCCCCAGGTCC-3′ and 5′-TGCCACAAACCCGTAGATCC-3′; and GAPDH: 5′-CTCATGACCACAGTCCATGC-3′ and 5′-TTCAGCTCTGGGATGACCTT-3′.

### 4.3. Flow Cytometry for Cancer Stem Cell Isolation and Drug Resistance Analysis

Cells were stained with CD44 antibody (phycoerythrin-conjugated; BioLegend, San Diego, CA, USA) or aldehyde dehydrogenase 1 (ALDH1; StemCell Technologies Inc., Vancouver, BC, Canada) followed by fluorescence-activated cell sorting analysis (FACS) to isolate the CSCs or examine their expression as previously described [30]. For drug resistance analysis, cells were stained with the ABCB5 antibody (Chemicon, Temecula, CA, USA) according to the manufacturer’s instructions. Fluorescence emission from 10,000 cells was measured with FACS Calibur (Becton Dickinson, San Jose, CA, USA) using CellQuest software.

### 4.4. Tumorspheres Culture for Cancer Stem Cell Selection

Spheroid cells from OSCC were plated in low attachment dishes at 10^4^ live cells/10-mm, and serum-free DMEM/F12 medium (Gibco, Grand Island, NY, USA) consisting of N2 supplement (Gibco), human recombinant basic fibroblast growth factor, and epidermal growth factor (R&D Systems, Minneapolis, MN, USA) was changed every other day until the tumorsphere formation was observed in about 2 weeks [31].

### 4.5. Knockdown of LINC00963

The pLV-RNAi vector was purchased from Biosettia Inc. (Biosettia, San Diego, CA, USA). The method of cloning the double-stranded shRNA sequence followed the manufacturer’s protocol. Oligonucleotide sequence of lentiviral vectors expressing shRNA that targets human LINC00963 was synthesized and cloned into pLVRNAi to generate a lentiviral expression vector. 

### 4.6. Analyses of Cancer Stemness Phenotypes

For migration or invasion capacities, 24-well Transwell system with an 8.0 μm porous transparent polyethylene terephthalate membrane was used. Cells (1 × 10^5^/well) were added to the upper chambers (filter coated with Matrigel for invasion assay) in the low serum medium. Medium supplemented with higher serum was used as a chemoattractant in the lower chamber followed by 24-hour incubation. Subsequently, cells that migrated through the membrane to the lower surface were stained with crystal violet and counted from 5 different fields under a fluorescence microscope. 

A soft agar colony-forming assay was carried out to evaluate the clonogenicity. Each well of a 6-well culture dish was coated with 2 mL bottom agar mixture (Sigma-Aldrich, St. Louis, MO, USA) (DMEM, 10% (v/v) fetal calf serum, 0.6% (w/v) agar). After the bottom layer was solidified, a top agar-medium mixture containing 2 × 104 cells was added and incubated at 37 °C for 4 weeks followed by crystal violet staining. The number of total colonies with a diameter ≥100μm was counted from 5 fields per well for a total of 15 fields in triplicate experiments [31]. Self-renewal capacity was assessed by serial sphere formation assay. Cell density was 1000 cells/mL in the serum-free medium as described earlier [24].

### 4.7. Cell Survival Assay

The 1 × 10^3^ cells were seeded in a 24-well plate, and then the MTT solution was added and incubated at 37 °C for 3 hours. The supernatant was removed, and 200 μL of dimethyl sulfoxide was added and then the OD value of the solution was analyzed by a microplate reader set at 570 nm. 

### 4.8. Subcutaneous Xenografts in Nude Mice

All the animal practices in this study were approved and in accordance with the Institutional Animal Care and Use Committee (IACUC) of Chung Shan Medical University, Taichung, Taiwan (CSMUH No: CS18043). Sh-Luc-expressing or Sh-LINC00963-expressing 1 × 10^4^ SAS-CSC with cisplatin treatment mixed with Matrigel (BD bioscience, San Diego, CA, USA) (1:1) were injected subcutaneously into BALB/c nude mice (6–8 weeks). Tumor volume (TV) was calculated using the following formula: TV (mm^3^) = (Length × Width^2^)/2.

### 4.9. Statistical Analysis

SPSS (version 13.0; SPSS, Inc., Chicago, IL, USA) was used for statistical analysis. Pearson’s correlation coefficient was used to evaluate the correlation between LINC00963 and stemness markers. Statistical differences were evaluated with the Student’s *t*-test and were considered significant at *p* < 0.05.

## 5. Conclusions

Collectively, the present study revealed that the elevated expression of LINC00963 in OSCC and oral CSCs facilitated malignant progression and drug resistance. Our results demonstrated that the LINC00963-mediated cancer stemness and resistance to chemotherapy were through ABCB5. We showed that suppression of LINC00963 may sensitize drug resistance and improve the treatment effect for OSCC patients.

## Supplementary Materials

The following are available online at https://www.mdpi.com/2072-6694/12/5/1073/s1, Figure S1: (A) Silencing LINC00963 decreased the expression of ABCB5 protein level in OSCC-CSC by western blotting. The amount of GAPDH protein of different crude cell extract was referred as loading control. (B) The whole blot (uncropped blots) of Supplementary Figure S1A. Table S1: Clinicopathological parameters in OSCC patients.
