# Supplementary material for: LINC00963 Promotes Cancer Stemness, Metastasis, and Drug Resistance in Head and Neck Carcinomas via ABCB5 Regulation
Source: Cancers (Basel). 2020 Apr 26;12(5):1073. doi: 10.3390/cancers12051073 (PMC7281373; doi:10.3390/cancers12051073)
Supplement: Supplementary file 1 [file cancers-12-01073-s001.pdf]

cancers

Article

LINC00963 Promotes Cancer Stemness, Metastasis, and Drug Resistance in Head and Neck Carcinomas via ABCB5 Regulation

Shiao-Pieng Lee, Pei-Ling Hsieh, Chih-Yuan Fang, Pei-Ming Chu, Yi-Wen Liao, Chuan-Hang Yu, Cheng-Chia Yu and Lo-Lin Tsai

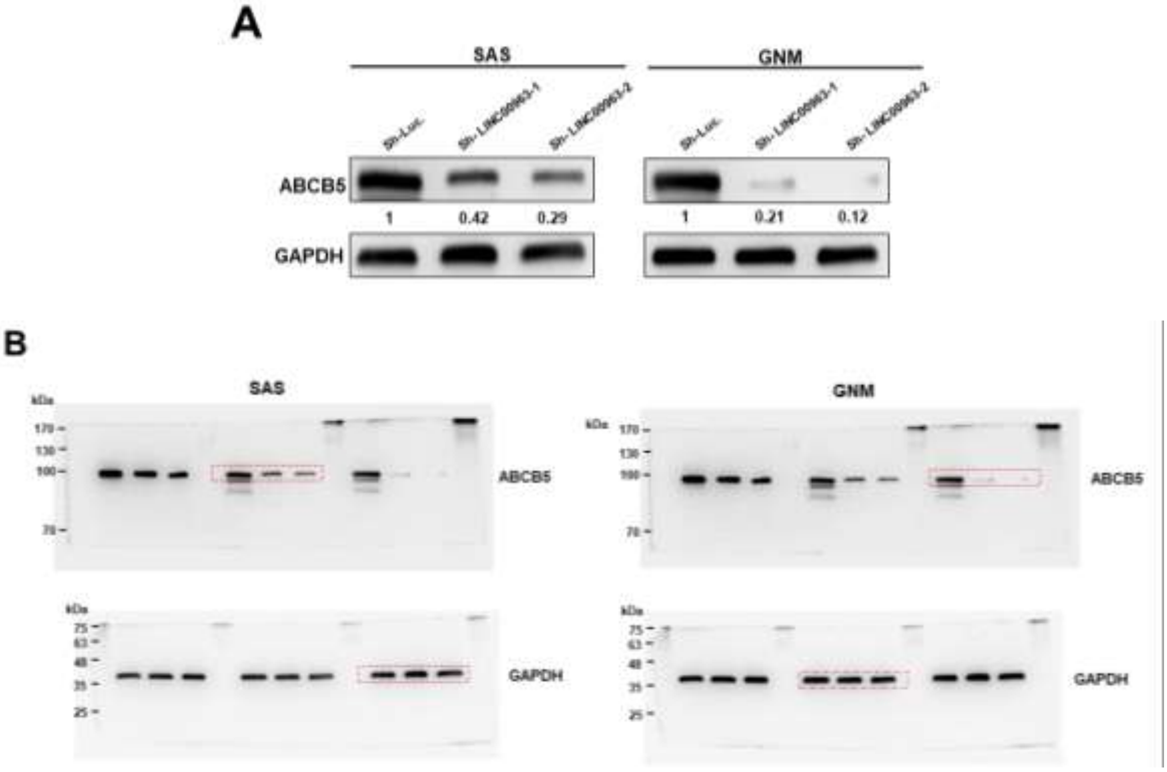

Figure S1. (**A**) Silencing LINC00963 decreased the expression of ABCB5 protein level in OSCC-CSC by western blotting. The amount of GAPDH protein of different crude cell extract was referred as loading control. (**B**) The whole blot (uncropped blots) of Supplementary Figure 1A.

Table S1. Clinicopathological parameters in OSCC patients.

**Parameters**	**Stage I and Stage II**	**Stage III and IV**
Age (years)	38-59	34-60
Mean age ± SD	45.5 ± 8.1	50.3 ± 8.2
Sex (M/F)	10/1	14/0
Site		
Buccal mucosa	4	4
Tongue	7	9
Other sites	0	1
Histopathologic diagnosis		
Well differentiation cancer	8	4
Moderate and Poor differentiation cancer	3	10
